# ﻿ *Polygonatumpraecox* (Asparagaceae), a new species from mid-eastern China revealed by morphological and molecular evidence

**DOI:** 10.3897/phytokeys.211.90456

**Published:** 2022-10-21

**Authors:** Yingfeng Hu, Yujun Liu, Maroof Ali, Wei Wu, Xiaohong Li, Longsheng Chen, Jianwen Shao

**Affiliations:** 1 College of Life Sciences, Anhui Normal University, Wuhu, Anhui 241000, China Anhui Normal University Wuhu China; 2 Anhui Academy of Science and Technology, Hefei 230000, China Anhui Academy of Science and Technology Hefei China; 3 Provincial Key Laboratory of Conservation and Utilization of Biological Resources, Wuhu, Anhui 241000, China Provincial Key Laboratory of Conservation and Utilization of Biological Resources Wuhu China

**Keywords:** flowering phenology, medicinal plant, *Polygonatumcaulialatum*, *P.cyrtonema*, *P.odoratum*

## Abstract

A new species, *Polygonatumpraecox* Y.F.Hu & J.W.Shao (Asparagaceae), is described and illustrated. This species is similar to *P.cyrtonema*, *P.odoratum* and *P.caulialatum*, but can be distinguished from *P.cyrtonema* by its racemose inflorescence, cylindrical hairless filaments and apex without a retrorse spur; from *P.odoratum* by its stout moniliform rhizome, straight stem and longer (1.7–2.2 cm long) floral tube; and from *P.caulialatum* by its upper part straight stem, yellowish-green corolla, lobes excurved and earlier flowering. The complete chloroplast genome of this new species is 155,115–155,256 bp in length. Phylogenetic analysis revealed that *P.praecox* is not genetically related to the above three morphological similar species, but is closely related to the two European species (*P.multiforum* and *P.latifolium*). This species is relatively common in mid-eastern China and has previously been confused with *P.cyrtonema*. As its wild resources have decreased in recent years due to over-exploitation for medicinal or edible purposes, we classify it as Near Threatened (NT) according to the IUCN Red List Criteria.

## ﻿Introduction

*Polygonatum* Mill., the largest genus of Tribe Polygonateae in Asparagaceae, contains more than 70 species that are widely distributed in the warm temperate, subtropical and boreal zones of the Northern Hemisphere (Tang 1978; Chen and Tamura 2000; Meng et al. 2014; Wang et al. 2016; Xia et al. 2022). The eastern Himalaya and Hengduan Mountains and also North East Asia are the centres of diversity where ca. 50 of the species occur (Floden 2017; Zhao et al. 2019; Xia et al. 2022). *Polygonatum* is one of the most important medicinal taxa in Asia, with some species being widely used in traditional Chinese medicine, such as *P.cyrtonema* Hua, *P.sibiricum* Redouté and *P.kingianum* Coll. & Hemsl. (Zhao et al. 2018; Chinese Pharmacopoeia Commission 2020; Fan et al. 2020; Li et al. 2021). Most species in this genus are edible and can be cultivated in forests without occupying farmland; thus, some of them are emerging as a new generation crop that offers high yield and nutrition, but do not require fertile land for growth (Si and Zhu 2021).

Accurate species delimitation has become of practical importance in conservation and utilisation of plant resources (Isaac et al. 2004). Flower features, especially filament shape and vestiture and its position in the perianth tube, are vital in *Polygonatum* species identification (Tamura 1991, 1993; Tamura et al. 1997; Floden 2012). However, the observation of these flower features is relatively difficult due to the short flowering period of most species, while the easily observed vegetative organ features show high plasticity in different habitats. The systematics and species classification of *Polygonatum* still requires study to understand the diversity as shown by the synonym lists for some species according to the Flora of China, such as *P.cyrtonema*, *P.odoratum* (Mill.) Druce and *P.kingianum* (Chen and Tamura 2000) and by the recent description of distinctive new species which have been published in recent years (e.g. Cai et al. 2015; Floden 2015; Yang et al. 2020; Chen et al. 2021).

During an investigation of wild germplasm resources of *Polygonatum* in eastern China, we made several collections of a possibly unknown plant with alternate leaves, thick moniliform rhizome and large yellow-green flowers (1.7–2.2 cm long). This plant is in appearance similar to and has usually been identified as *P.cyrtonema*. However, we found that this plant differs from *P.cyrtonema* in its filaments (inserted near the distal 1/3 of the perianth tube, hairless and apical part without saccate-convex), flowering phenology (mid-March to early April) and inflorescence type (racemose). After further observation of its morphology and flowering phenology, chloroplast sequencing and phylogenetic analysis, we identified it as a new species which has been overlooked. Thus, we report the results of our investigation and the new species, named as *Polygonatumpraecox* Y.F.Hu & J.W.Shao, sp. nov., is described and illustrated here.

## ﻿Materials and methods

### ﻿Morphological assessment

**Figure 1.**
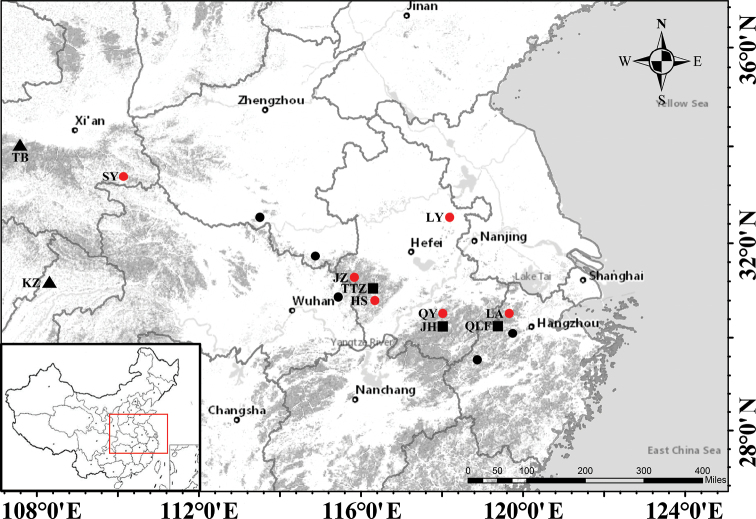
Distribution map of *Polygonatumpraecox* (red dots showing locations found in this study; black dots showing locations identified by specimen examination), *P.caulialatum* (black triangle) and *P.cyrtonema* (black square).

Six populations of the putative novel species (*Polygonatumpraecox*) were found in Anhui, Zhejiang and Shaanxi Provinces (Fig. 1, Table 1) and some individuals from three populations (JZ, LY and QY) were transplanted to the Botanical Garden in Anhui Normal University for further observation of their morphology and flowering phenology. Some populations of *P.cyrtonema* (TTZ, QLF and JH, Table 1), *P.odoratum* (QS and CZ, Table 1) and *P.caulialatum* (KZ and TB, Table 1) were also collected and transplanted to the Garden for further morphological assessment. In the field, more than five living plants in each population were randomly selected for rhizomes, leaves, flowers morphological observations. The stability and variation patterns of these morphological traits (especially the filaments characteristics) and the flowering period were further observed in transplanted populations. All voucher specimens were deposited at the Herbarium of Anhui Normal University (ANUB). The specimens of *Polygonatum* in Herbaria PE, CSH, K, KUN, WU, JSPC, XBGH and NAS were also examined through digital platforms (CVH, GBIF, NSII), with special attention on the type specimens of *P.cyrtonema* and its synonyms and morphological similar species.

**Table 1.** The information of the sampled and investigated populations.

***Polygonatumpraecox* (putative new species)**				
LY	Langya Mountain Scenic Spot, Langya, Chuzhou City, Anhui Province	32.2777	118.2866	ON736440
LA	Qingliangfeng Mountain, Linan, Hangzhou City, Zhejing Province	30.1451	118.8705	ON943064
JZ	Tiantangzhai Scenic Spot, Jinzhai, Liuan City, Anhui Province	31.1256	115.7718	ON736439
SY	Jiashi Gorge, Banyan Town, Shanyang, Shangluo City, Shaanxi Province	33.3181	109.7701	ON736441
QY	Wumei Village, Yangtian Town, Qingyang, Chizhou City, Anhui Province	30.5829	117.9702	
HS	Bancang Nature Reserve, Huoshan, Anqing City, Anhui Province	31.1147	116.1091	
***P.caulialatum***				
KZ	Bashan Grand Canyon Scenic Area, Kaizhou, Chongqing City	31.6505	108.4345	ON943065
TB	Qingfengxia Forest Park, Taibai, Baoji City, Shaanxi Province	34.0099	107.4407	
***P.odoratum***				
QS	Tianzhu Mountain Scenic Spot, Qianshan, Anqing City, Anhui Province	30.7392	116.4663	
CZ	Langya Mountain, Langya, Chuzhou City, Anhui Province	32.2792	118.2811	
***P.cyrtonema***				
TTZ	Tiantangzhai Scenic Spot, Jinzhai, Liuan City, Anhui Province	31.1256	115.7718	
QLF	Qingliangfeng Mountain, Linan, Hangzhou City, Zhejing Province	30.1451	118.8706	
JH	Jiuhua Mountain Scenic Spot, Qingyang, Chizhou City, Anhui Province	30.5112	117.8448	

### ﻿Genome sequencing, assembly and annotation

Fresh leaves of one individual per population in five populations (SY, JZ, LY, LA of *P.praecox* and KZ of *P.caulialatum*, Table 1) were collected and dried in silica for molecular analysis. Genomic DNA was extracted using Tiangen DNAsecure Plant Kit (DP320). All libraries were built through optimisation of the construction process and sent to the Germplasm Bank of Wild Species in Southwest China (GBOWS, Kunming, China) for Illumina sequencing. Approximately 3 GB of raw data were generated for each sample. Raw data were trimmed by removing adapters and low-quality reads by FastQC/Trimmomatic. The complete chloroplast genome was assembled using Getorganelle v.1.7.5.2, through the original data reads (fastq / FQ file) with K-mer = 21, 65 and 105 (Jin et al. 2020). The plastome gene sequences of *P.odoratum* (MZ150858) were adopted as reference and seed sequences. PGA (Qu et al. 2019) was used for plastome annotation with manually checking the start/stop codons in Geneious 10.2.3 (http://www.geneious.com). The plastid genome map was drawn using OGDRAW (Greiner et al. 2019). Basic characteristics of chloroplast genomes were read in Geneious (Table 2).

**Table 2.** Basic characteristics of chloroplast genomes of *Polygonatumpraecox*, sp. nov.

Characteristic	*Polygonatumpraecox*	*P.caulialatum*
Total length (bp)	155,115–155,256	155,318
GC%	37.7%–37.7%	37.7%
LSC length (bp)	84,252–85,225	84,252
SSC length (bp)	18,450–18,474	18,462
IR length (bp)	26,318–26,323	26,302
Total genes	112	112
Protein-coding genes	76	76
rRNA genes	4	4
tRNA genes	32	32

### ﻿Phylogenetic analyses

In order to reveal the phylogenetic relationship of the putative novel species and its related species, plastome sequence data of *Polygonatum* and outgroup (*Heteropolygonatumogisui*) from GenBank were downloaded (Floden and Schilling 2018; Xia et al. 2021, 2022; Wang et al. 2022). All sequences were aligned using MACSE v.2 and one of the inverted repeats (IRa) was deleted using Geneious (e.g. Ranwez et al. 2018) before further analysis. The phylogenetic tree was constructed using Maximum Likelihood (ML) methods and Bayesian Inference (BI) methods. The best substitution model was determined by ModelFinder in PhyloSuite (Kalyaanamoorthy et al. 2017; Zhang et al. 2020). The ML analysis was performed using IQ-TREE v.1.6.12 with 1000 bootstrap replicates and (GTR) + G +I (Nguyen et al. 2015). The BI analysis was conducted in MrBayes v.3.2 (Ronquist et al. 2012). The Markov Chain Monte Carlo (MCMC) algorithm was run for 20 million generations and the trees were sampled every 1000 generations. Convergence was determined by examining the average standard deviation of the split frequencies (< 0.01). The first 25% of calculated trees were discarded as burn-in and the remaining trees were used to construct a consensus tree to estimate the posterior probability (PP).

## ﻿Results and discussion

### ﻿Characteristics of the complete plastid genome

**Figure 2.**
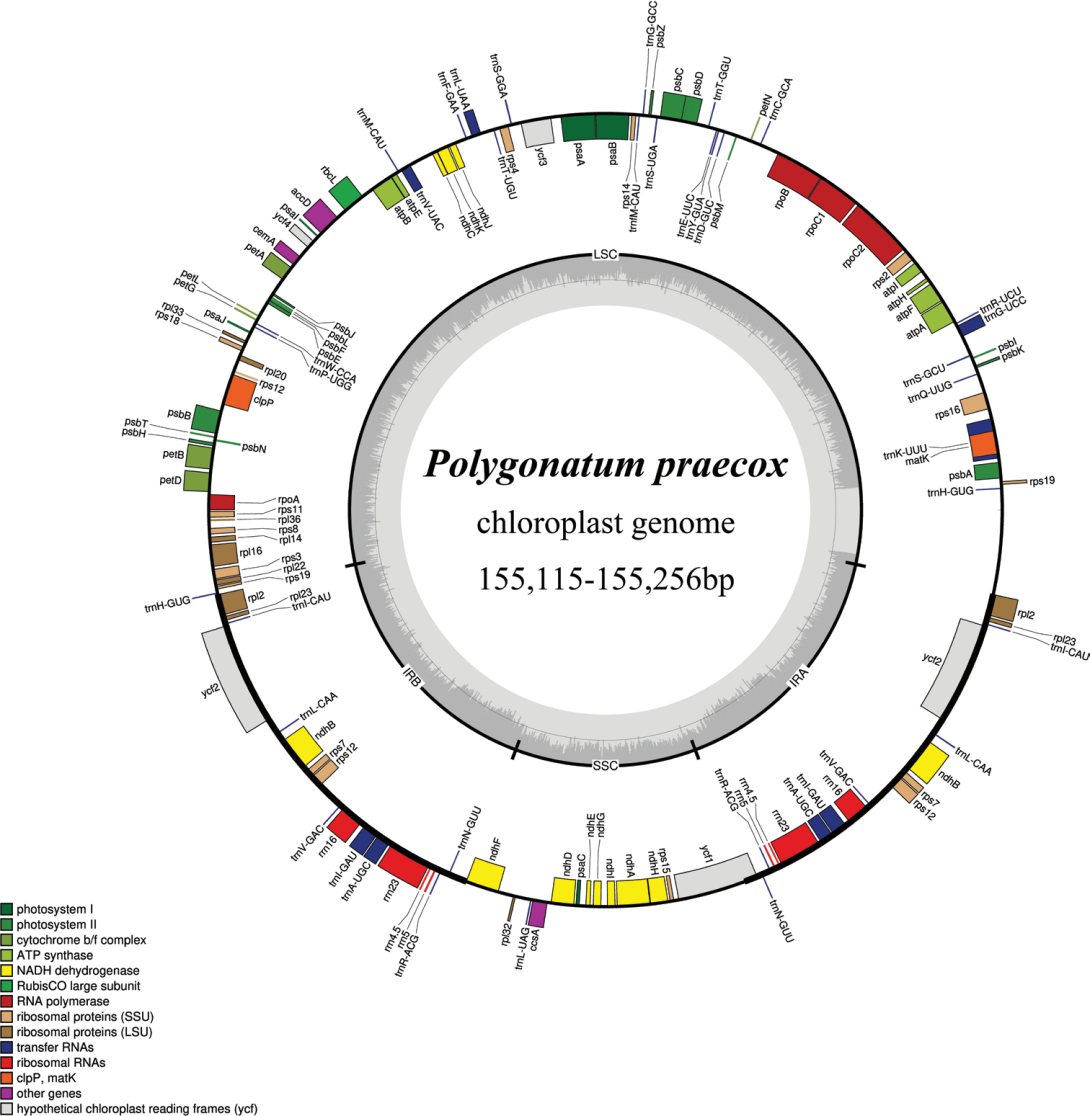
Plastid genome map of *Polygonatumpraecox* Y.F.Hu & J.W.Shao, sp. nov.

The length of chloroplast complete genome of *Polygonatumpraecox* samples was 155,115–155,256 bp (Fig. 2) and *P.caulialatum* was 155,318 bp; both possessed typical quadripartite structure (IRa, IRb, LSC and SSC). The characteristics and statistics of their plastid genomes are summarised in Table 2.

### ﻿Phylogenetic relationship

**Figure 3.**
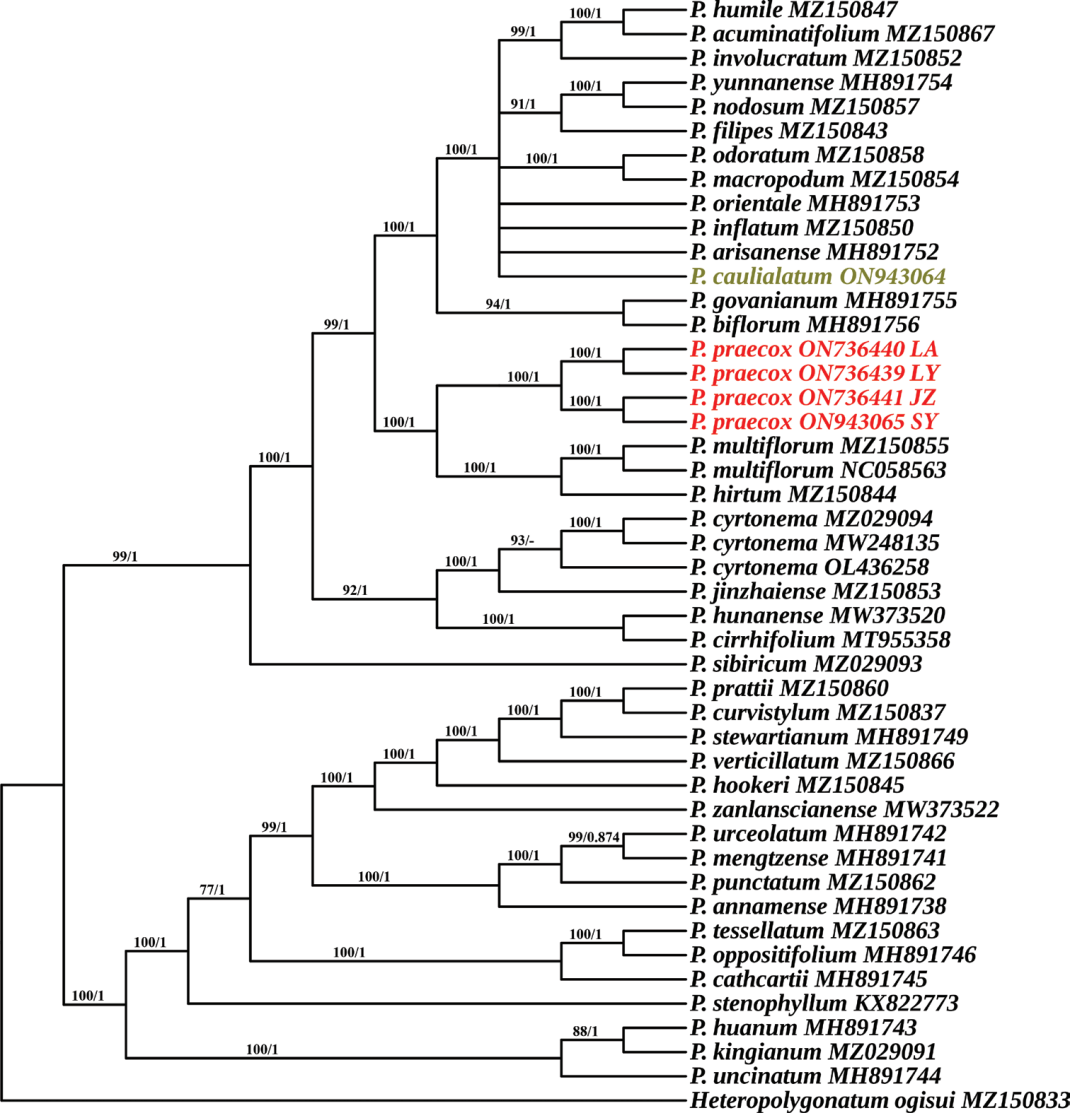
Phylogenetic relationships of the new species and its related species in *Polygonatum*, inferred from Maximum Likelihood (ML) and Bayesian Inference (BI) methods, based on the plastid genomes without one of the inverted repeats (IRa). Numbers on the branches indicate the bootstrap support of the ML and the posterior probability of BI analyses. The phylogenetic position of *P.praecox* is highlighted in red and *P.caulialatum* in brown. GenBank accession numbers are displayed after the species name.

As in previous phylogenetic analyses of *Polygonatum* (Meng et al. 2014; Xia et al. 2022), three well-supported clades corresponding to monophyletic sections were also resolved, i.e. *Verticillata*, *Sibirica* and *Polygonatum* (Fig. 3). The four individuals of the putative novel species from different geographic populations grouped into a monophyletic clade (Bootstrap Support (BS) = 100%, Bayesian Inference (BI) = 1), which were not sister to the three morphologically similar species (*P.cyrtonema*, *P.odoratum* and *P.caulialatum*, Fig. 3), although they are all in section Polygonatum. Unexpectedly, the putative novel species is closely related to the two European species (*P.multiforum* Kunth and *P.latifolium* (Jacq.) Desf.) (Fig. 3).

### ﻿Morphological assessment

**Figure 4.**
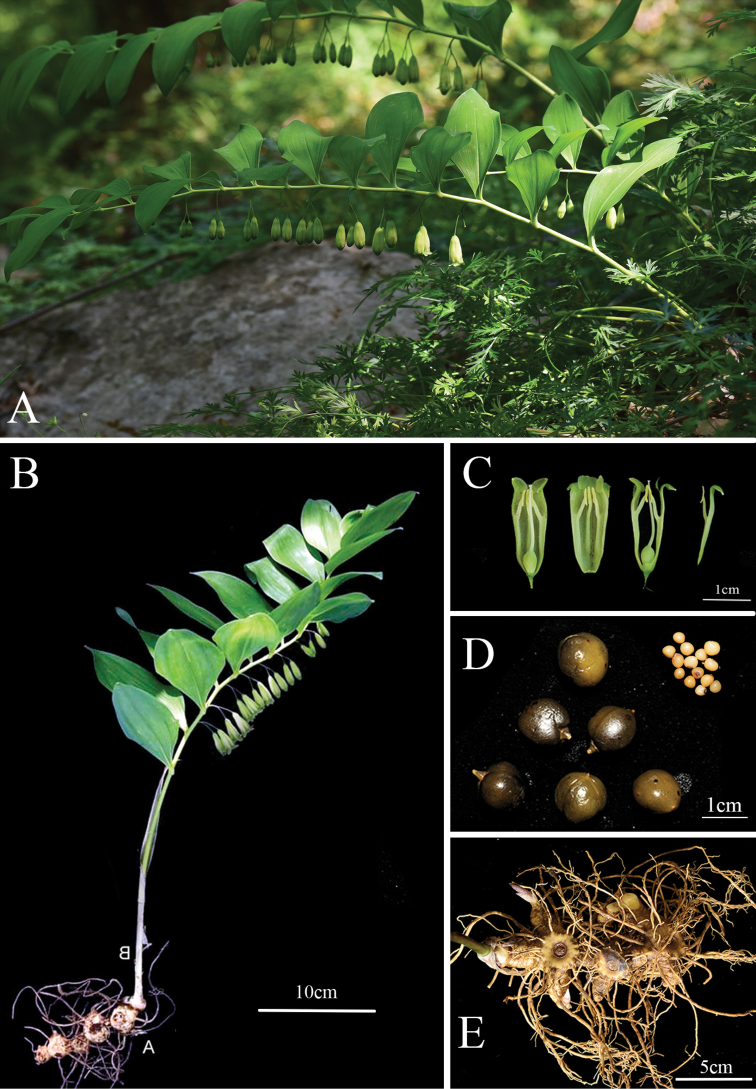
*Polygonatumpraecox* Y.F.Hu & J.W.Shao, sp. nov. **A** habitat **B** general habit **C** longitudinal section of floral tube, showing stamens and pistil **D** seeds and fruits, soaked in alcohol **E** rhizome with roots. All Photos by Yingfeng Hu.

**Figure 5.**
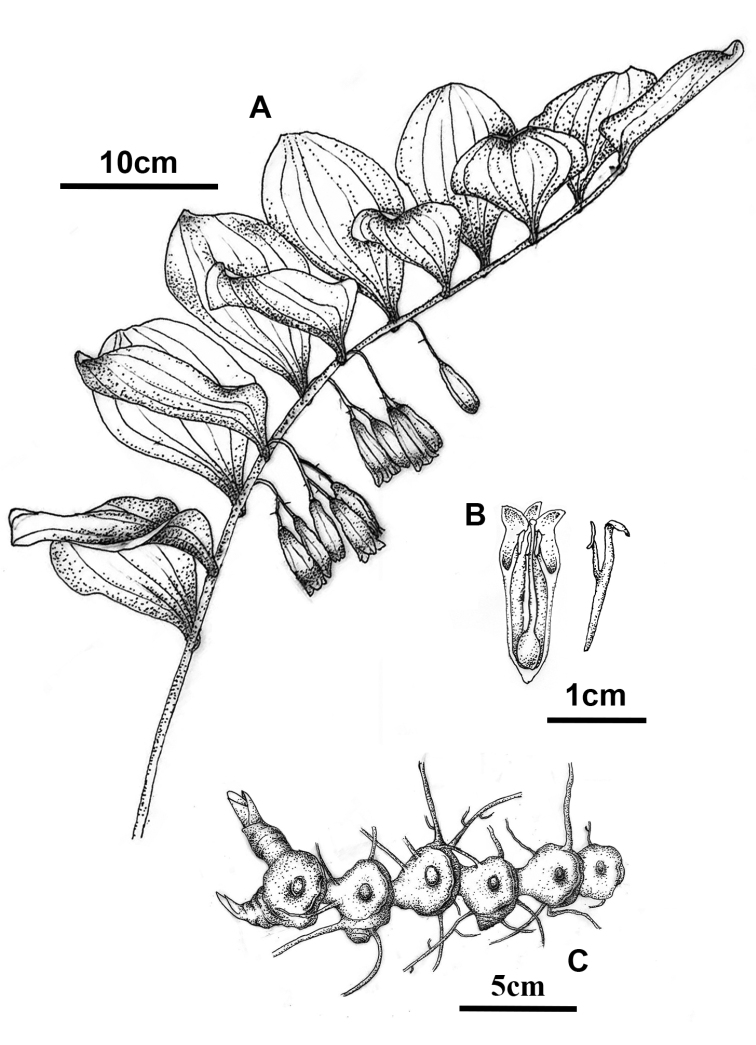
Illustration of *Polygonatumpraecox* Y.F.Hu & J.W.Shao, sp. nov. **A** morphology of aboveground part **B** longitudinal section of floral tube, showing stamens and pistil **C** moniliform rhizome. Drawn according to the holotype by Wei Wu.

The new species is morphologically similar to *Polygonatumcyrtonema* and *P.caulialatum* in its alternate oblong leaves, thick moniliform rhizome and large flowers (corolla about 1.7–2.2 cm long) (Figs 4, 5, Table 3), but it differs from *P.cyrtonema* in racemose inflorescence (vs. umbel-like), cylindrical and hairless filaments and its apex without saccate-convex (vs. papillose or shortly cottony, apex slightly dilated or with saccate-convex) and flowering from mid-March to early April (vs. from late April to late May); it differs from *P.caulialatum* in its terete stem (vs. obviously angled in upper part), yellowish-green corolla and lobes excurved (vs. green-white and lobes not excurved) and earlier flowering period (mid-March to early April vs. May to June). As to inflorescence type and flowering phenology, *Polygonatumpraecox* is also similar to *P.odoratum* (raceme inflorescence and flowering from mid-March to early April), but they are very different in rhizome morphology (moniliform vs. terete) and stem (terete vs. angled). In morphology, this new species is also easily distinguished from its genetically related species *P.multiforum* and *P.latifolium* by its moniliform rhizome (vs. terete) and campanulate-cylindrical yellowish-green floral tube (vs. cylindrical, but somewhat contracted in the middle and whitish). In summary, there are obvious differences between the new species and its related species in morphology, especially in filament traits. However, because of the short flowering period, most previously collected specimens of *Polygonatum* were without blooming flowers and the stamen morphology is not easy to observe on dry specimens, which may be the reason for this new species being neglected for a long term.

**Table 3.** Morphological differences amongst *Polygonatumcyrtonema*, *P.odoratum*, *P.caulialatum* and *P.praecox*.

	*P.cyrtonema*	*P.odoratum*	*P.caulialatum*	*P.praecox*
Rhizome	usually gingeriform, 1–2.5 cm thick	terete, 0.5–1.0 cm thick	moniliform, 1.5–2.5 cm thick	moniliform, 1.5–2.5 cm thick
Stem	50–100 cm, terete	20–60 cm, upper part angled	40–80 cm, upper part angled	40–80 cm, terete
Inflorescence	umbel-like, 2–7(–14) flowered	raceme, 1–3(–5) flowered	raceme, 1–2(–3) flowered	raceme, 1–3(–4) flowered
Filament	papillose or shortly cottony, apex slightly dilated or saccate-convex	cylindrical and extending inwardly, smooth or verruculose	cylindrical and extending inwardly, smooth and glabrous	cylindrical and extending inwardly, smooth and glabrous
Corolla	yellowish-green, lobes excurved	green-white, lobes slightly excurved	green-white, lobes not excurved, crown slightly constricted	yellowish-green, lobes excurved
Flower phenology	late April to late May	mid-March to early April	May to June	mid-March to early April

### ﻿Taxonomic treatment

#### ﻿ Polygonatum praecox

Taxon classificationPlantaeAsparagalesAsparagaceae

Y.F.Hu & J.W.Shao sp. nov.

2FD17FC9-278E-59B0-9A97-1BFEA1ACF8B9

urn:lsid:ipni.org:names:

Figs 4 , 5 , 6

##### Type.

**Figure 6.**
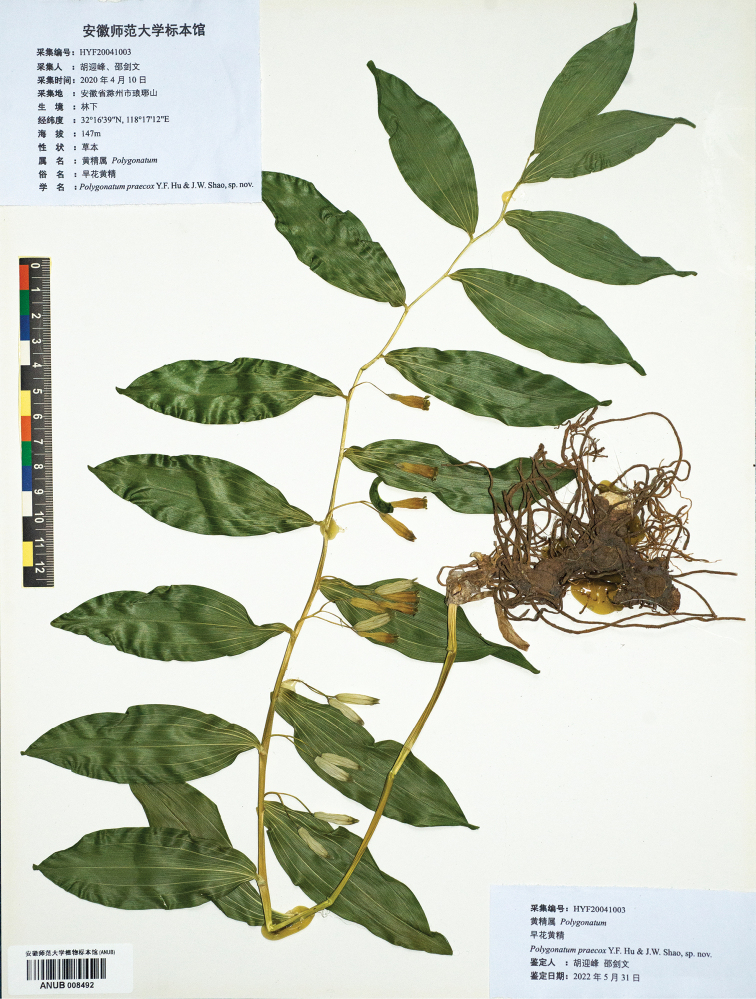
Holotype of *Polygonatumpraecox* Y.F.Hu & J.W.Shao, sp. nov.

China. Anhui: Chuzhou City, Langya District, Langya Mountain, 32°16'39"N, 118°17'12"E, Altitude: 147 m, 10 Apr 2020, *Yin Feng Hu & Jian Wen Shao* HYF20041003 (holotype: ANUB, 008492, Fig. 6; isotypes: ANUB, 008491, 008493).

##### Diagnosis.

Most similar to *P.cyrtonema*, but differs in racemose inflorescence, cylindrical and glabrous filaments and apex not saccate convex.

##### Description.

Rhizome moniliform, rarely tuberous moniliform, 1.5–2.5 cm thick. Stem arching, 40–80 cm, glabrous and not angled. Leaves 14–22, alternate; petiole short or nearly sessile; leaf blade elliptic to oblong-lanceolate, 8–13 × 4–6 cm, apex usually acuminate. Inflorescences raceme, (1)2–3(4)-flowered; peduncle 1–2 cm; bracteoles borne on the middle part of pedicel, subulate, < 2 mm or absent. Flowers pendulous, pedicel 0.5–1.5 cm long. Perianth yellowish-green, campanulate-cylindrical, 1.7–2.2 cm long; lobes ca. 3 mm long, excurved. Filaments inserted near the distal 1/3 of the perianth tube, cylindrical and extending inwardly, 3–6 mm long, smooth, apex without saccate-convex. Anthers 3.5–4.0 mm long. Ovary 4–6 mm in diam.; style 1.2–1.5 cm long. Berries black, ca. 1.2–1.5 cm in diam., 9–15 seeded.

##### Phenology.

Flowering from mid-March to early April and fruiting from May to September.

##### Etymology.

The specific epithet *praecox* alludes to early flowering of the new species as compared to *Polygonatumcyrtonema*, a morphologically similar species. The Chinese name of the new species is here given as 早花黄精 (Zǎo huā huáng jīng).

##### Distribution and habitat.

Currently, *Polygonatumpraecox* is known from more than 10 populations and it is fairly widely distributed in middle-eastern China (Fig. 1). This species often occurs near valley streams under forest shade and on gravel or soil with good water permeability between elevations of 50 m to 1200 m.

##### Additional specimens examined

**(paratypes).** China. Anhui: Langya District, Langya Mountain, alt. 200 m, 3 Jul 1964, *Anonymous*, 101383 (JSPC); Langya District, Langya Mountain, 4 May 1957, *Teng Yan Chang*, 0305591 (KUN). Zhejiang: Linan County, Changhua, alt. 1080 m, 17 Jun 1957, *Deng Lin Bing* 00223701 (NAS); Linan County, Tianmu Mountain, 18 May 1955, *Yuan Chang Qi* 00553413 (NAS). Hubei: Yinshan County, Wujiashan Forest Farm, alt. 1070 m, 06 Apr 2015, *Chen Bin* 0092527 (CSH); Yinshan County, alt. 815 m, 26 Apr 2015, *Ge Bin Jie* 0092551 (CSH). Henan: Song County, Xihe River, 8 May 1972, *Anonymous*, 00223667 (PE); Neixiang County, Baotianman Nature Reserve, 28 Aug 2008, *Liu Meng Ya* 0003911 (HEAC). Shaanxi: Shanyang County, Jiashi Gorge, Banyan Town, 26 July 2009, *Li En Feng* 010008 (XBGH).

##### Conservation status.

Near Threatened. *Polygonatumpraecox* is relatively common in middle-eastern China. As it is similar to *P.cyrtonema* in morphology and these two species occasionally co-exist in the wild, this new species is usually recognised as *P.cyrtonema* and has been exploited for medicinal or edible purposes. Its wild resources have clearly decreased in recent years. Therefore, we classify it as Near Threatened (NT) according to the IUCN Red List Criteria (IUCN 2019).

## Supplementary Material

XML Treatment for Polygonatum praecox
